# A Broadband Tunable Terahertz Metamaterial Absorber Based on Single-Layer Complementary Gammadion-Shaped Graphene

**DOI:** 10.3390/ma13040860

**Published:** 2020-02-14

**Authors:** Fu Chen, Yongzhi Cheng, Hui Luo

**Affiliations:** School of Information Science and Engineering, Wuhan University of Science and Technology, Wuhan 430081, China; zs101141@163.com (F.C.); luohui0112@163.com (H.L.)

**Keywords:** terahertz metamaterial absorber, graphene, broadband tunable absorption, surface plasmon polaritons

## Abstract

We present a simple design of a broadband tunable metamaterial absorber (MMA) in the terahertz (THz) region, which consists of a single layer complementary gammadion-shaped (CGS) graphene sheet and a polydimethylsiloxane (PDMS) dielectric substrate placed on a continuous metal film. The Fermi energy level (*E_f_*) of the graphene can be modulated dynamically by the applied DC bias voltage, which enables us to electrically control the absorption performance of the proposed MMA flexibly. When *E_f_* = 0.8 eV, the relative bandwidth of the proposed MMA, which represents the frequency region of absorption beyond 90%, can reaches its maximal value of 72.1%. Simulated electric field distributions reveal that the broadband absorption mainly originates from the excitation of surface plasmon polaritons (SPPs) on the CGS graphene sheet. Furthermore, the proposed MMA is polarization-insensitive and has wide angles for both transverse-electric (TE) and transverse-magnetic (TM) waves in the broadband frequency range. The broadband absorption capacity of the designed MMA can be effectively adjusted by varying the Fermi energy level of graphene. Lastly, the absorbance of the MMA can be adjusted from 42% to 99.1% by changing the *E_f_* from 0 eV to 0.8 eV, which is in agreement with the theoretical calculation by using the interference 41theory. Due to its simple structure and flexible tunability, the proposed MMA has potential application prospects in tunable filtering, modulators, sensing, and other multispectral devices.

## 1. Introduction

Terahertz wave metamaterial absorbers (MMAs) have recently attracted great attention and become one of the widespread research hotspots due to their promising applications in communication, imaging, detection and sensing, and stealth [1,2,3,4,5]. In the past few years, a great number of terahertz MMAs based on various resonator structures have been proposed to achieve single narrowband, dual or multi-narrowband, and broadband absorption [6,7,8,9,10,11,12]. MMAs exhibiting high absorption performance are usually realized by periodic array structures consisting of lossy materials that can effectively confine and consume the incident electromagnetic (EM) fields [1,13,14]. However, most of the reported MMAs suffer from some factors that restrict their practical applications, such as the fixed operating frequency range and the absorption level. In those studies, the absorption properties of MMAs turned out to be strongly dependent on the established geometries of the unit cell structure, which cannot be altered after fabrication is completed. The urgently needed dynamic control methods for MMAs still demand prompt solutions. Graphene, as a dense two-dimensional (2D) film material consisting of monolayer carbon atoms packed into honeycomb crystal lattice, can be regarded as a promising candidate material because of its unique properties, including optical transparency, electron mobility, and excellent mechanical properties [15,16,17,18]. The most attractive property is that the permittivity and conductivity of graphene can be dynamically tuned by changing the Fermi energy through chemical doping or external bias voltage, achieving dynamically tunable MMAs [19,20,21]. 

In the last few years, MMAs based on graphene have received increasing interest and have achieved tremendous progress. Various MMAs based on patterned graphene structures, such as disks [21], ribbons [22,23], patches [24], cross fishnets [25], and other microstructures [26,27,28,29,30,31], have been proposed to enhance the absorption. It is believed that the perfect absorption of the graphene-based MMA is mainly originated from excitation of surface plasmon resonances (SPRs) in the periodic patterned graphene structures. However, most of these MMAs are usually narrowband, which greatly limits their applications in some circumstances. Numerous efforts have been made to realize multi- and broadband absorption by various methods, including multilayer graphene structures [32], multiple graphene resonators in a unit cell [33], structured graphene with gradually changing geometric sizes [34], and other hybrid-patterned metal-graphene composite structures [33,34,35,36,37]. Although the broad absorption bandwidth of the MMAs can be enhanced, most of them still suffer from some drawbacks, including difficulty with absorption tuning via biasing, significant polarization and incident angle dependence, and complicated fabrication technology. Thus, although great progress has been made in the field of MMA, there is still plenty of room for improvement. A novel polarization-insensitive and wide-angle MMA based on a simple graphene structure still remains to be further investigated.

In this work, we propose a novel MMA based on a periodic patterned graphene structure, which can achieve polarization-insensitive and wide-angle broadband terahertz absorption with flexibly active tunability. The proposed tunable MMA is composed of a single-layer periodical complementary gammadion-shaped (CGS) graphene array placed over a polydimethylsiloxane (PDMS) dielectric substrate deposited on a gold film. First, the structure design of the proposed terahertz MMA is demonstrated. Then, the absorption mechanism of the proposed MMA is clarified by analyzing the field distributions and multireflection interference theory. Next, the absorption characteristics of the proposed MMA are analyzed by varying the oblique incident angles of terahertz waves and the Fermi energy level of the graphene as well. The calculated results based on the multireflection interference theory model are in agreement with simulated results. Numerical results indicate that the relative bandwidth, which presents the frequency range of absorption beyond 90%, can reach its maximum value of 72.1% when *E_f_* = 0.8 eV. Both simulation and calculation results reveal that the MMA maintains excellent absorption performance when the incident angle is below 50° for both transverse electric (TE) and transverse magnetic (TM) polarized terahertz waves. By controlling the graphene Fermi energy from 0 eV to 0.8 eV, the absorbance of the MMA can be easily tuned from 42% to 99.1%. Hence, the proposed tunable MMA based on graphene is believed to have promising applications in terahertz sensing, detection, imaging, and cloaking.

## 2. Structure Design, Theory, and Simulations

**Figure 1:**
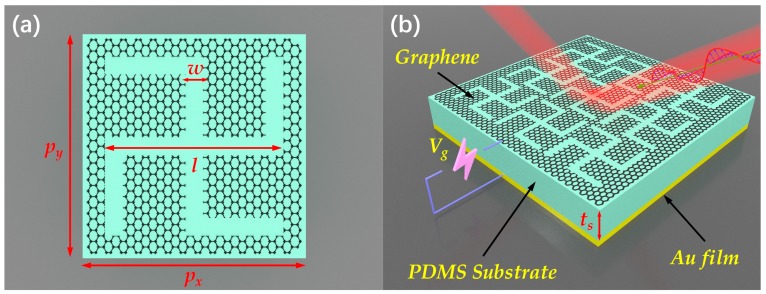
The scheme of designed tunable metamaterial absorber (MMA): perspective views of (**a**) the unit cell structure and (**b**) the two-dimensional array.

The design scheme of the proposed terahertz tunable MMA is illustrated in Figure 1. In most of the reported cases, the graphene patterns in MMAs are discrete, which would lead to great obstacles for practically fabrication and electrical control of graphene based MMAs. Hence, the design of a complementary graphene structure is a feasible strategy in order to tune the conductivity of graphene via extra bias voltage, allowing the flexible electrical control of MMAs. As shown in Figure 1a, the unit cell of the proposed MMAs has been designed to be a CGS graphene sheet with a typical non-mirror symmetric. The entire proposed MMA possess a typical sandwich structure composed of a single-layer periodical CGS graphene resonator on top of a dielectric PDMS substrate, which is deposited on a continuous metallic film, as shown in Figure 1b. Generally, the periodical CGS graphene can be produced by large-scale graphene synthesis, transfer, and etching techniques. The CGS graphene patterns on the dielectric substrate layer can be created by using electron beam lithography [38,39]. Thus, the overall unit cell structure is polarization-insensitive for the normal incidence terahertz waves due to its uniaxial four-fold (C_4_) rotational symmetry, and the graphene conductivity of the MMA can be controlled by applying a DC voltage *V*_g_. 

The graphene is numerically modelled by an effective medium with a certain thickness *t*_g_, whose relative permittivity can be calculated by *ε*_g_ = 1 + i*σ*_g_/(*ε*_0_*ωt*_g_) [28,38], where *σ*_g_ is the graphene surface conductivity, *ε*_0_ is the vacuum permittivity constant, and *ω* is the angular frequency of the incident terahertz wave. In this study, we assume an effective medium with a thickness of *t*_g_ = 1 nm to replace the three-layer graphene sheets [29], since the thickness of the mono-layer graphene sheet is 0.34 nm [40,41,42]. The complex surface conductivity *σ*_g_ of graphene can be modelled as a thin surface sheet and characterized by the Kubo formula [29,43,44]: *σ*_g_ = *σ*_intra_ + *σ*_inter_, where *σ*_intra_ and *σ*_inter_ denote intra-band and inter-band conductivities, respectively. In view of the Pauli exclusion principle, the inter-band contribution of graphene conductivity can be neglected in low terahertz and far-infrared regions [45]. Thus, in the terahertz range of interest, the graphene conductivity *σ*_g_ is only determined by intra-band contribution *σ*_intra_, which can be simplified and approximately described as [29]:

(1) $$\sigma_{g}(\omega )\approx \sigma_{\mathrm{intra}}(\omega )=j\frac{e^{2}K_{B}T}{\pi \hbar^{2}(\omega +j\tau^{-1})}(\frac{E_{f}}{K_{B}T}+2\ln(\exp(-\frac{E_{f}}{K_{B}T})+1))$$

where *ω*, *E_f_*, *τ*, and *T* are the radian angular frequency of the incident terahertz wave, chemical potential (Fermi energy), relaxation time, and environmental temperature, respectively. The relaxation time *τ* characterizes the plasmon decay on account of impurities, which is fixed to 0.1 ps, and *T* is the kelvin temperature, which is fixed to 300 K in this study. In addition, *e* represents the charge of an electron, *ℏ* is the reduced Planck’s constant, and *k_B_* is the universal constant representing the Boltzmann constant. The approximate estimated theoretical relation between *E_f_* and *V*_g_ can be expressed as [30,45]:

(2) $$E_{f}=\hbar v_{f}\sqrt{\frac{\pi \varepsilon_{0}\varepsilon_{r}V_{g}}{et_{s}}}$$

where *v_f_* is the Fermi velocity, *V*_g_ is the extra bias voltage, and *ε*_0_ and *t_s_* are the effective relative permittivity and thickness of the middle dielectric layer, respectively. According to the equation above, it can be found that the absorption property of the proposed MMA can be controlled via bias voltage between the CGS graphene layer and the continuous metallic film. The continuous metallic film of the MMA is formed with gold, with the relative permittivity obtained from the Drude model [46]. Since the thickness of the continuous gold film is much larger than the typical skin depth in the terahertz region, the transmission wave can be largely suppressed. Polydimethylsiloxane (PDMS) with a relative permittivity and loss tangent of 2.35 and 0.06, respectively, was used as the dielectric spacer layer [47]. The final optimized geometric parameters of the unit cell structure are given as: *p_x_* = *p_y_* = 25 μm, *l* = 24 μm, *w* = 2.5μm, *t_s_* = 14 μm. The unit cell structure of the proposed MMA was set to be periodical along the *x-* and *y*-axis with periods of 25 μm to avoid diffraction at the normal incidence rate for frequencies up to 12 THz. In this configuration, the surface plasmon polaritons (SPRs) of the single-layered CGS graphene can be excited, therefore it makes sense to utilize the resonant absorption characteristics of graphene surface plasmon polaritons (SPPs) to enhance the absorption of incident terahertz wave [29,48].

To verify the absorption efficiency of the proposed MMA, three-dimensional full wave numerical simulations were performed in CST Microwave Studio based on the finite element method (FEM). In simulation, the periodic boundaries in both *x*- and *y*-axis directions (*x*-*y* plane) are used, and incident terahertz waves are set to propagate along the *z*-direction, as shown in Figure 1b. Floquet ports for the incident terahertz waves in the z-direction are assigned to the unit cell, so that both TE and TM mode polarizations can be easily obtained. For TE or TM mode polarization, the incident wave vector *k* is set to be in the *xoz* or *yoz* plane and the electric field is in the *x*-axis direction (TE) or in the *y*-axis direction (TM). The absorbance of the proposed MMA can be calculated by the formula *A*(*ω*) = 1 − *T*(*ω*) − *R*(*ω*) = 1 − |*S*_21_(*ω*)|^2^ − |*S*_11_(*ω*)|^2^, where *T*(*ω*) = |*S*_21_(*ω*)|^2^ and *R*(*ω*) = |*S*_11_(*ω*)|^2^ are transmittance and reflectance, respectively. In our design, the transmittance is zero (*S*_21_(*ω*) = 0), owing to the thickness of the bottom continuous gold film, which is greater than the skin depth across the whole terahertz region. Thus, the absorbance can be simplified as *A*(*ω*) = 1 − |*S*_11_(*ω*)|^2^. 

## 3. Results and Discussion

**Figure 2:**
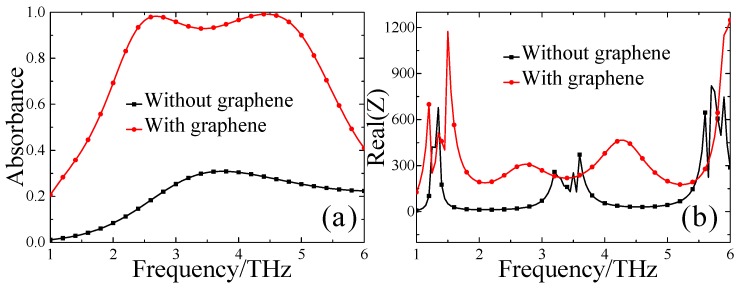
(**a**) The simulated absorbance of the designed MMA with and without graphene, and (**b**) the corresponding real part of the wave impedance.

Firstly, the absorbance performances of MMAs with and without the CGS graphene sheet under normal incident terahertz waves were investigated, and the simulated results are depicted in Figure 2. Here, in the simulation we assumed the absolute temperature *T* = 300 K, relaxation time of graphene *τ* = 0.1 ps, and the initial Fermi energy *E_f_* = 0.8 eV. As shown in Figure 2a, for the structure with the CGS graphene sheet, an effective absorption band (the frequency range of which possesses absorbance beyond 90%) from 2.31 THz to 5.01 THz can be obtained, and the corresponding relative bandwidth is up to 72.1%. In addition, absorption peaks reach up to 98.2% and 99.1% at 2.7 THz and 4.4 THz, respectively. On the other hand, the absorbance peak barely reaches 30.8% when the CGS graphene sheet is removed from the MMAs. 

To better understand the operation principle of the proposed MMA structure, we calculated the wave impedances of the MMA with and without the CGS graphene sheet from the simulated reflection coefficients according to the S-parameter retrieval method [49]. On average, the real part of the wave impedance of the MMA with the CGS graphene sheet is close to the free space impedance (Real(Z) ≈ 377 Ω) from 2.31 THz to 5.01 THz, whereas the one without the CGS graphene sheet evidently deviates from 377 Ω in the frequency range of interest. Thus, it can be concluded that the proposed MMA with the CGS graphene sheet can be tuned to approximately impedance-match the free space in the broadband frequency range, consequently leading to a relatively wide effective absorption band.

**Figure 3:**
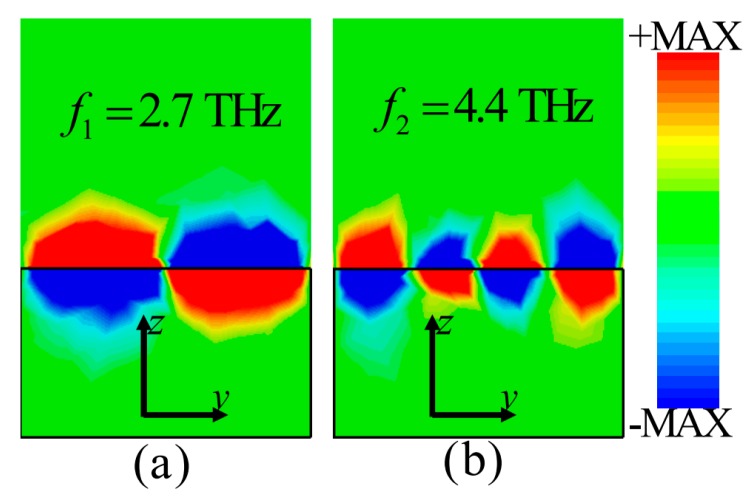
The electric field (*E*_z_) distributions of the *y*–*z* plane of the unit cell structure at (**a**) *f*_1_ = 2.7 THz and (**b**) *f*_2_ = 4.4 THz, respectively (*E_f_* = 0.8 eV).

To reveal the physical mechanism of the proposed MMA based on CGS graphene, an intuitive and straightforward method is to investigate the electric field distributions in resonance. As shown in Figure 3, the z-component (*E_z_*) of the electric field distributions on the *y*-*z* plane of the unit cell structure at absorption peak frequencies of 2.7 THz and 4.4 THz, respectively, are illustrated. It can be found that the *z*-component (*E_z_*) of the electric field is mainly concentrated near the interface between the CGS graphene sheet and the PDMS substrate at 2.7 THz and 4.4 THz, respectively. The tight field confinement around the CGS graphene sheet on the top layer can be observed clearly, which means that all the electric fields are trapped and subsequently dissipated on the graphene layer. The strong terahertz wave absorption supposedly caused by excitation of different resonance modes. In Figure 3a,b, it can be seen that the two observed absorption peaks essentially originate from the fundamental and second order graphene SPP resonances, respectively. In addition, it can also be seen that the electric fields near the interface area between the graphene and PDMS substrate are composed of propagating waves related to the far-field interaction and evanescent waves, which correspond to the near-field interaction effect [50,51,52]. In summary, it can be concluded that the stronger electric field confinement characteristics of the unit cell structure above are consistent with the absorbance spectra shown in Figure 2a.

**Figure 4:**
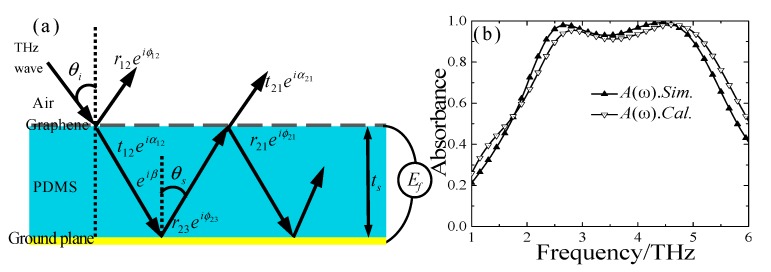
(**a**) Illustration of the interference model of the proposed MMA, and (**b**) the simulated and calculated absorbance of the designed MMA when *E_f_* = 0.8 eV.

To further reveal physical insights from the proposed graphene-based MMA, quantitative analysis based on interference theory was employed in this work. Interference theory based on Fabry-Pérot-like resonance cavity can provide a profound understanding of the underlying physics for the proposed MMA. According to interference theory, the MMA can be considered as a Fabry-Pérot-like resonance cavity, which would induce multiple EM interference effects in multireflection in the incident terahertz waves [10,11,12,13,14,53]. The superposed multiple reflections would then destructively interfere with the direct reflection from the air-CGS graphene sheet and would finally result in high-level absorption [53]. As shown in Figure 4a, the multiple reflections in the interference model include two interfaces: the air-periodical CGS graphene sheet interface and the PDMS substrate-ground gold film interface. Here, since the thickness of the PDMS is large enough, the near-field coupling effect and magnetic response between the CGS graphene sheet and the ground plane can be neglected. As shown in Figure 4a, when the incident terahertz wave interacts with the CGS graphene sheet, one part of the wave is reflected in the air-CGS graphene interface and the other part emits from the interface after multiple reflections and transmissions. Thus, the overall reflection coefficient of the MMA is the superposition of the multiple reflections, which can be expressed as follows [13,53]:

(3) $${\tilde{r}}_{all}=r_{12}e^{i\phi_{12}}-\frac{t_{12}t_{21}e^{i(\alpha_{12}+\alpha_{21}+2\tilde{\beta })}}{1+r_{21}e^{i(\phi_{21}+2\tilde{\beta })}}$$

where $\tilde{\beta }=\beta_{r}+i\beta_{i}=-\sqrt{\varepsilon_{r}}k_{0}t_{s}/\cos(\theta_{s})$ is the propagation phase; *ε_r_* and *t*_s_ are the relative permittivity and thickness of PDMS substrate, respectively; *k*_0_ is the free space wave number; and $\theta_{s}=\arcsin[\sin\theta_{i}/\sqrt{\varepsilon_{r}}]$ is calculated with arbitrary incidence angle *θ_i_*. In addition, ${\tilde{r}}_{12}=r_{12}e^{i\phi_{12}}$ and ${\tilde{t}}_{12}=t_{12}e^{i\alpha_{12}}$ denote the reflection and transmission coefficient at the air-CGS graphene interface, respectively. At the CGS graphene-PDMS interface, the corresponding reflection and transmission coefficients can be represented by ${\tilde{r}}_{21}=r_{21}e^{i\phi_{21}}$ and ${\tilde{t}}_{21}=t_{21}e^{i\alpha_{21}}$, respectively. The reflection and transmission coefficients (${\tilde{r}}_{12}$,${\tilde{r}}_{21}$,${\tilde{t}}_{12}$, and ${\tilde{t}}_{21}$) of the CGS graphene-PDMS interface can be obtained via FEM simulation. Therefore, the total absorbance can be retrieved through the formula $A(\omega )=1-|{\tilde{r}}_{{}_{all}}{|}^{2}$. Figure 4b shows the calculated absorbance of the proposed MMA when the Fermi energy is set to be *E_f_* = 0.8 eV, which is in good agreement with the simulated result. This result indicates that the interference theory based on the Fabry-Pérot-like resonance cavity can be used to legitimately explain the physics mechanism of the proposed MMA.

**Figure 5:**
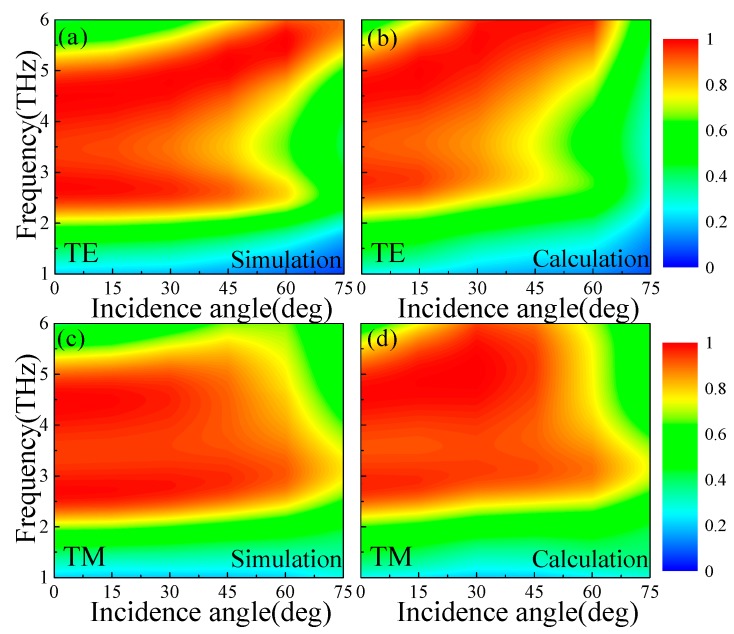
The (**a**,**c**) simulated and (**b**,**d**) calculated absorbance of MMAs under different oblique incidence angles for (**a**,**b**) TE and (**c**,**d**) TM waves (*E_f_* = 0.8 eV).

For practical application, the absorption performance of the proposed MMA should be robust to the different polarization angles and oblique incident angles for both TE and TM waves. Obviously, the proposed terahertz MMA is polarization-independent for both TE and TM waves due to the uniaxial four-fold (C_4_) rotational symmetry of the unit cell structure (not show). Here, we consider the absorption performance of the proposed MMA (*E_f_* = 0.8 eV) under different oblique incident angles for both TE and TM mode polarization waves. Both the simulated and calculated absorbance under different oblique incidence angles for TE and TM waves are depicted in Figure 5, which are in good agreement with each other.

As shown in Figure 5a,b, for TE waves, it can be seen that broadband absorption performance can be maintained up to 45°. Beyond 45°, the broadband absorption performance will be decreased gradually, especial for the lower frequency range. This is because the incident magnetic flux between the CGS graphene sheet and ground plane will decrease with the increase of incidence angle [10]. In addition, the frequency range of the stronger absorption has a slight blue shift with the increase of the oblique incidence angle. For TM waves, as shown in Figure 5c,d, the high-level absorption of the broadband frequency can be maintained up to 65°. This means that the magnetic flux between the CGS graphene sheet and ground plane is nearly unchanged at a larger incidence angle for TM waves [10]. Meanwhile, the absorption spectra of the proposed MMA also display a slightly blue shift with increasing incidence angles for the TM wave. These results indicate that the proposed MMA can maintain the absorption stability with different polarization angles and wide incident angles for both TE and TM waves.

**Figure 6:**
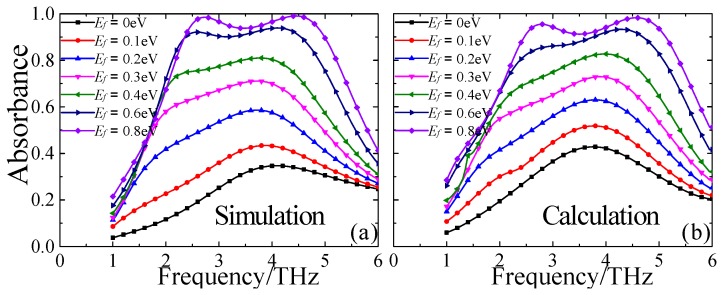
The (**a**) simulated and (**b**) calculated absorbance spectra under normal incident TE waves with different Fermi energy levels (*E_f_*).

Furthermore, the electrically tunable properties of the broadband terahertz MMA were also investigated numerically. Graphene, as one kind of tunable photoelectric material, is often applied to regulate the absorption amplitude and frequency range of a MMA. The surface conductivity of a graphene sheet relates primarily to its Fermi energy, which can be easily controlled by applying bias voltage or electrostatic chemical doping [33,34,35]. Here, the Fermi level (*E_f_*) of the CGS graphene sheet placed on the top layer of the MMA structure is adjusted dynamically by changing the external bias voltage *V*_g_, as shown in Figure 1b. The absorption properties of the proposed MMA were discussed when the Fermi level (*E_f_*) of the CGS graphene sheet was continuously tuned from 0 to 0.8 eV through external bias voltage. Figure 6a,b present the simulated and calculated absorption spectra as a function of terahertz frequency and the Fermi level (*E_f_*) under normal incident TE waves. It is obvious that the absorption level is enhanced gradually with the increasing *E_f_*, while the operating frequency range is nearly unchanged. Moreover, the simulated average absorbance increases from 42% to 99.1% gradually with the increment of *E_f_* from 0 to 0.8 eV, while the operating frequency range remains nearly unchanged, which is ell consistent with the calculation result based on interference theory. Thus, it can be concluded that the proposed MMA with flexible tunability could be used as a tunable broadband spatial amplitude modulator or attenuator in terahertz regimes. In addition, as distinguished from the previous MMAs consisting of isolated graphene disks, ribbons, and multi-resonators, the proposed MMA, which possesses a net CGS graphene layer with inherent continuous electrical connection, may greatly simplify the previously redundant gating structures to just one voltage gate and realize the flexible tunability of the absorption properties. The design strategy of this tunable broadband terahertz MMA can be easily scaled to infrared or visible regimes if the dimensions are reduced to nano- or even lower scales.

## 4. Conclusions

In conclusion, we have demonstrated a new efficient route for achieving a broadband terahertz tunable MMA using a net CGS graphene sheet deposited on a PDMS dielectric spacer supported by a metallic reflecting plate. Simulation results indicate that the relative bandwidth of the proposed MMA, which represents a frequency region of absorption beyond 90%, reaches its maximal value of 72.1% when *E_f_* = 0.8eV. The simulated electric field distribution of one unit cell structure reveals that the stronger broadband absorption of the MMA mainly originates from the continuous excitation of the fundamental and second order graphene SPP resonances. Additionally, multiple-reflection interference theory was used to quantitatively analyze the absorption performance of the designed MMA with wide-range incidence angles and different Fermi levels (*E_f_*). The theoretical calculations and full-wave simulations are in excellent agreement with each other. The MMA has been proven to be insensitive to the polarization states of incident terahertz waves, and the absorbance remains at 80% with incident angles up to 45° for TE waves and up to 65° for TM waves. Compared to conventional MMAs consisting of isolated graphene disks, ribbons, multi-resonators, or multi-layered structures, the continuous net CGS graphene structure greatly simplifies the electrostatic gating structure to achieve flexible tunability. By controlling the Fermi level (*E_f_*) via external bias voltage of the graphene sheet, the absorbance can be tuned continuously from 42% to 99.1%. This work offers a new perspective on the simple design of graphene-based tunable terahertz broadband MMAs. Benefitting from these promising properties, including the simple structure, absolute polarization insensitivity, wide incident angle, and the flexible tunability of the broadband absorption, the proposed MMA could be engineered for various applications in broadband terahertz spatial amplitude modulators, attenuators, sensors, and other optoelectronic devices.
